# Ubiquinol attenuates γ-radiation induced coronary and aortic changes via PDGF/p38 MAPK/ICAM-1 related pathway

**DOI:** 10.1038/s41598-023-50218-w

**Published:** 2023-12-27

**Authors:** Walaa A. El-Sabbagh, Noha A. Fadel, Rania M. El-Hazek, Ahmed H. Osman, Laila A. Ramadan

**Affiliations:** 1 Drug Radiation Research Department, National Center for Radiation Research and Technology (NCRRT), Egyptian Atomic Energy Authority, Cairo, Egypt; 2 Department of Pathology, Faculty of Veterinary Medicine, Cairo University, Giza, Egypt; 3 Pharmacology and Toxicology Department, Faculty of Pharmacy, Egyptian Russian University (ERU), Cairo, Egypt

**Keywords:** Cell adhesion, Cell signalling, Biochemistry, Drug discovery

## Abstract

Endothelial vascular injury is one of the most pivotal disorders emerging during radiotherapy. It is crucial to rely on strong antioxidants to defend against vascular damage. The current study was carried out to investigate the ameliorative effect of ubiquinol (Ubq) against gamma (γ)-radiation induced aortic and coronary changes, with highlighting its role in suppression of p38 mitogen activated protein kinase (MAPK). Exposure to γ-radiation was adopted as a potent detrimental model that induces vascular tissue damage. Concisely, male albino rats were irradiated at a dose level of 7 Gy and treated daily with Ubq (10 mg/kg/day, p.o.) for 7 days pre-and post-irradiation. At the end of the experiment, lipid profile, 8-hydroxydeoxyguanosine (8-OHdG), gene expression of intercellular adhesion molecule (ICAM-1), platelet derived growth factor (PDGF), p38 MAPK and matrix metalloproteinase-9 (MMP-9) were estimated. Exposure to radiation significantly deteriorates aortic and coronary tissues. Conversely, administration of Ubq significantly reduced serum t-cholesterol, LDL and triglycerides (*p* = 0.001). In addition, Ubq prevented oxidative DNA damage (8-OHdG) (*p* = 0.1) and reduced serum MMP-9 (*p* = 0.001) which contributed to the endothelial cells damage. The positive impact of Ubq was more apparent in suppression of both PDGF (*p* = 0.001) and p38 MAPK (*p* = 0.1) protein concentrations, leading subsequently in reduction of ICAM-1 (*p* = 0.001) gene expression. As a conclusion, vascular endothelial damage brought on by γ-radiation is one of the leading causes of coronary and aortic deteriorations which could be successfully mitigated by Ubq.

## Introduction

Radiotherapy (RT) is a cornerstone of cancer treatment, with > 50% of cancer patients now undergoing therapeutic radiation. Although RT efficiently destroys cancer cells, it might lead to emergence of cardiovascular consequences, including alterations in the cardiac vasculature, coronary spasm and atherosclerosis^1^. Previous experimental and clinical studies have demonstrated the detrimental effects of radiation exposure on the coronary and aortic arteries, as well as the molecular routes implicated in such adverse consequences^2–5^.

Exposure to ionizing radiation (IR) induced DNA damage and oxidative stress molecules, which play a significant role in the endothelial cells injury since they are linked to the activation of inflammatory and apoptotic pathways^4,6,7^. The endothelial injury is assumed to be initiated with the infiltration of monocytes into the intima^8^, which is then followed by the inclusion of low density lipoproteins (LDL) and the development of fatty streaks^9^. Oxidation of LDL is usually associated with surging of inflammatory cytokines. This triggers the activation of nuclear transcription factor-kappa beta (NF-κβ) which in-turn up-regulates the transcription of adhesion molecules; selectin-E, intracellular adhesion molecule-1 (ICAM-1), and vascular cell adhesion molecule-1 (VCAM-1). The association between ICAM-1and incidences of cardiovascular diseases has been previously established^10^. In detailed, ICAM-1 is expressed on the surface of endothelial cells in response to tissue damage and inflammation^11^. It regulates the leukocyte recruitment from circulation to sites of inflammation and control the leukocyte rolling and adhesive interactions with the vessel wall^12,13^. This ultimately led the oxidative state to rise, resulting in the production of oxidized LDL particles and induction of several atherogenic factors, which induce the formation of atherosclerotic plaque^14^. Besides, the significance of growth factors, such as platelet-derived growth factor (PDGF), in the development of neointima has been established in animal models of arterial damage^15^; PDGF promotes vascular smooth muscle cells (VSMCs) proliferation, migration, and vascular intimal thickening^16^.

Moreover, one of the signaling pathways, that are highly implicated in the endothelial injury induced post radiation, is the activation of the mitogen-activated protein kinases (MAPKs)^9^. The MAPKs are family of central signaling molecules that respond to numerous stimuli by phosphorylating a variety of substrates, among which is p38 proteins which play an important role in the endothelial activation, as it is involved in the process of neutrophil adherence to the endothelial cells^17^. Furthermore, matrix metalloproteinases (MMPs) are a significant contributor to endothelial injury; after radiation, the amount of MMPs is up-regulated, which leads to the degradation of extracellular matrix components^9^.

Thus, interfering with such signaling pathway and controlling the induced inflammatory and oxidative state of endothelial cells may represent a potential therapeutic strategy where the vascular cellular function can be preserved or treated against radiation detrimental impact.

A tremendous amount of interest has reemerged towards substances that exhibit potential anti-oxidant and anti-inflammatory activities with less adverse effect during radiotherapy. CoQ10 is endogenously biosynthesized substance which present ubiquitously in all tissues. It exists predominantly in two forms, reduced form as ubiquinol (Ubq) and oxidized form as ubiquinone. Ubq is predominant in the mammalian body and has more bioavailability compared to ubiquinone^18^. Clinically, it is notable that endogenous production deficiency contributes to the pathogenesis of several disorders, since its biosynthesis is markedly diminished with ageing^19^. In particular, Ubq was previously endowed with major antioxidant activity in the vascular compartment by preventing LDL oxidation and ameliorating the endothelial dysfunction associated with dyslipidemia^20^. The role of Ubq in modulating endothelial dysfunction has been linked with its capacity in reducing oxidative stress and inflammatory markers^21^. In addition, previous researches validated the significant impact of CoQ10, the oxidized form of Ubq, on the endothelium and vascular system, which in turn influences the incidence, etiology, and progression of other CVDs such as coronary artery disease^22^.

Based on the aforementioned background, the current study was performed to validate the ability of Ubq to antagonize the radiation-induced coronary and aortic harm, in an attempt to investigate the molecular mechanism underlying its vascular protective effect, using a rat model of aortic and coronary injury induced by gamma radiation, and showing the crosstalk between oxidative and inflammatory pathways.

## Results

### Pilot histopathological examinations

In the current study, Ubq treatment strategy depended on the histopathological outcomes of aortic arch and coronary artery after exposure to γ-radiation. Two groups of rats were exposed to 7 Gy acute dose of γ-radiation, the aortic arches and coronary arteries of the 1st group (IR1) were examined after 1 week following irradiation, and those in the 2nd group (IR2) were examined after 2 weeks.

**Figure 1:**
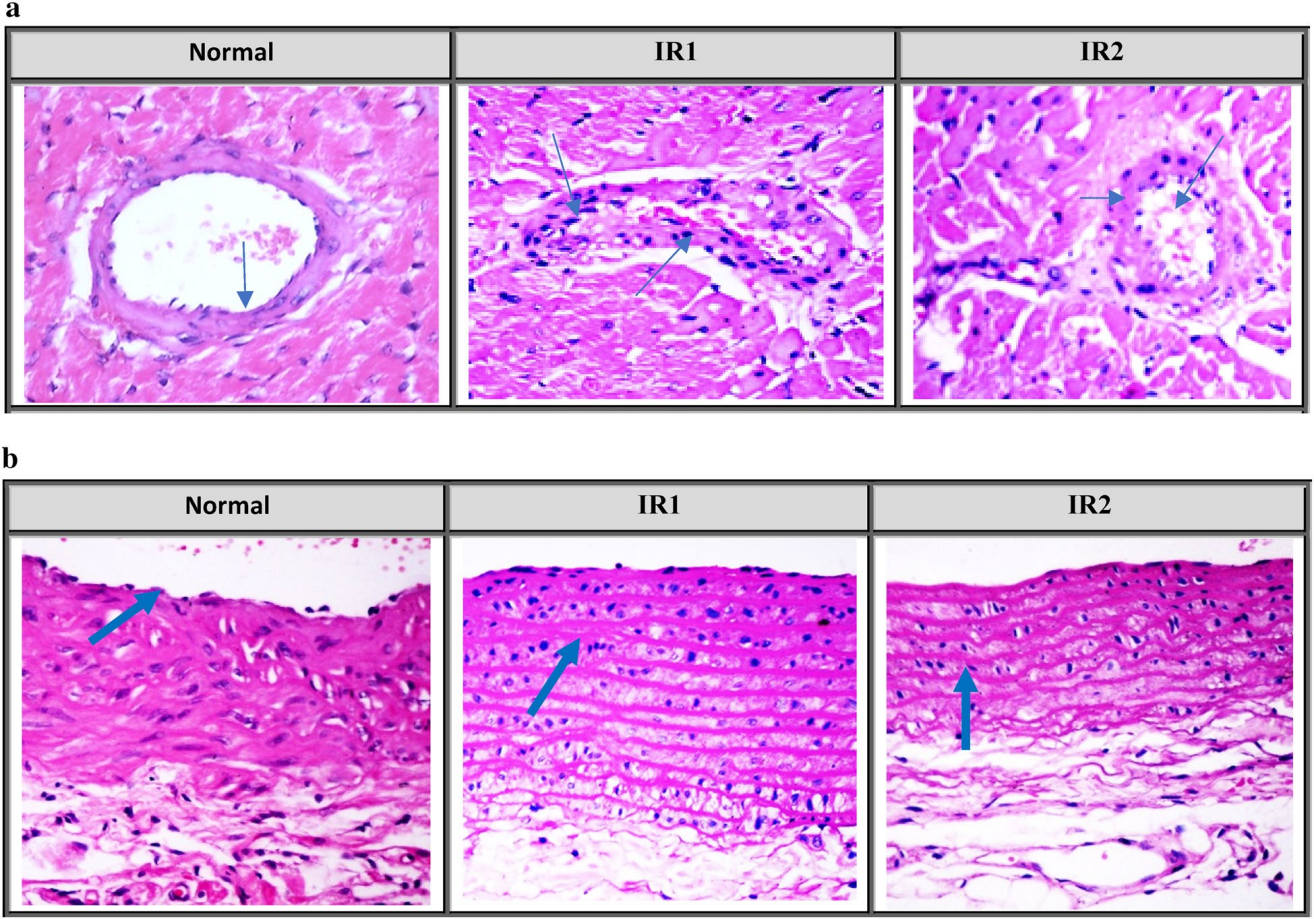
(**a**) Photomicrograph of coronary arterial branch showing normal histological structure with intact endothelial lining (arrow) in Normal group. Damaged endothelial cells with foam cells and leukocytic infiltration (arrow) were shown in IR1 group, while IR2 group showing thickening of its wall with swelling and protrusion of endothelial lining (arrow) (H&E × 400). (**b**) Photomicrograph of aortic tissue section showing endothelial cells lined on the tunica intima (arrow) in Normal group, increased wall thickness and leukocytic infiltration (arrow) in IR1 group, while a mild edema in-between elastic bands (arrow) was shown in IR2 group (H&E × 400).

The coronary artery of control normal group demonstrated normal histological structure with intact endothelial lining (Fig. 1a). IR1 showed endothelial cells damage and accumulation of fat in the form of foam cells infiltration. Vasculitis was seen in the majority of examined coronary branches which were characterized by perivascular edema and leukocytic infiltration, mainly lymphocytes and macrophages (score 4) (Fig. 1a). Animals in the IR2 group showed the same histological findings as the previous group, in addition to a marked thickening in the coronary branch wall with swelling and protrusion of endothelial lining (score 4) (Fig. 1a).

Aortic tissue section obtained from Normal rats group displayed normal histological structure with unremarkable changes (Fig. 1b). Moreover, aortic tissue section of IR1 group showed an increase in wall thickness accompanied by increased density of number of elastin bands. Also, endothelial degeneration, necrosis and disorientation of smooth muscle cells were seen. Multifocal mononuclear cells infiltration in the aortic wall with marked edema of tunica intima were seen (score 3) (Fig. 1b). On the other hand, the aortic tissue section of IR2 group revealed nearly the same histological findings as the previous group with less thickness and mild edema in-between elastic bands (score 2) (Fig. 1b).

Accordingly, the 7-day period was chosen to further investigate the effects of Ubq based on structural alterations in the aorta and coronary arteries after acute radiation exposure.

In order to achieve the optimum conservative impact on vascular tissue, two treatment strategies were tested on two groups of irradiated (7 Gy) rats; 1st group was administered Ubq (10 mg/kg) for 7 days post radiation, and the other group received Ubq (10 mg/kg) for 14 days [7 days prior to irradiation and 7 days following irradiation].

**Figure 2:**
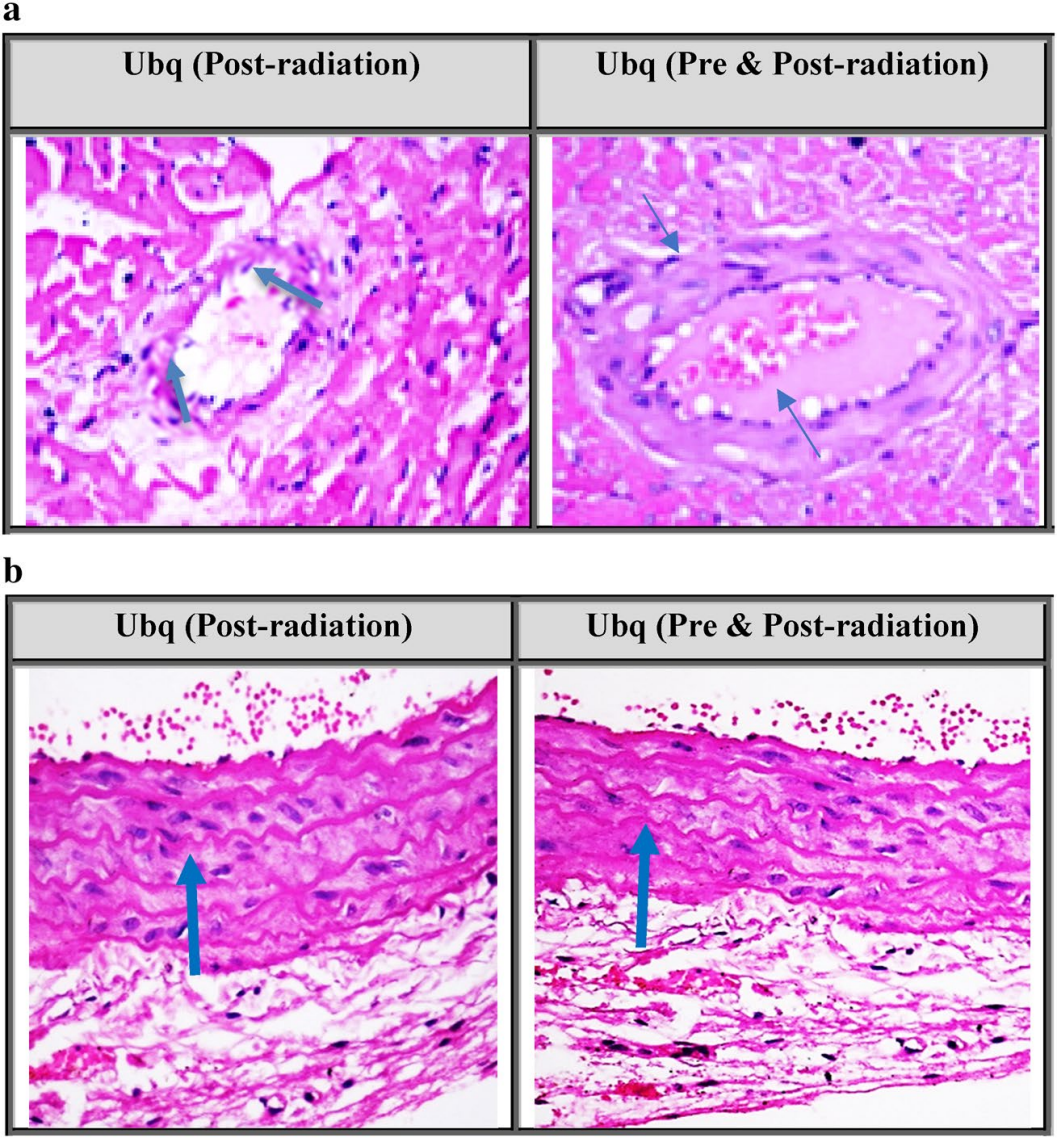
(**a**) Photomicrograph of coronary arterial branch showing swelling of endothelial ling with few leukocytic infiltration (arrow) in Ubq (Post-radiation) group and showing intact endothelial lining with mild edema of coronary wall (arrow) in Ubq (Pre and Post-radiation) group, comparable with IR non-treated group (H&E × 400). (**b**) Photomicrograph of aortic tissue section showed reduction of aortic wall thickness with less mononucleolar cells infiltration (arrow) in Ubq (Post-radiation) group. Mild edema in-between elastic bands with few leukocytic infiltration (arrow) was shown in Ubq (Pre and Post-radiation) group, comparable with IR non-treated group (H&E × 400).

Administration of Ubq for 7 days post-radiation showed less severe lesion, swelling of endothelial cells with few leukocytic infiltrations and perivascular edema in the coronary arterial branch, as compared to the irradiated non-treated group (score 2) (Fig. 2a). On the other side, the group that received Ubq for 14 days (7 days prior to irradiation and 7 days following irradiation) showed more improvement in the coronary arterial branch represented by intact endothelial lining with mild edema of the coronary wall and few numbers of fat droplets deposited on the wall (score 1) (Fig. 2a).

As for the aortic tissue section, the post-radiation treated group showed improvement as a reduction in aortic wall thickness with less mononucleolar cells infiltration (score 1) (Fig. 2b). While, the pre-and post-radiation treated group displayed more improvement, revealed by mild edema in-between elastic bands, with few leukocytic infiltration (score 1) (Fig. 2b). As a result, the administration of Ubq pre- and post- radiation was selected in the main study.

### Total lipid profile

As shown in Table 1, exposure to γ-radiation led to significant increments in serum t-cholesterol, TGs, VLDL and LDL by 106.76%, 100.2%, 67.35% and 62.34%, respectively, as compared to control group (*p* = 0.001). Interestingly, daily treatment with Ubq (10 mg/kg) [pre- and post- radiation exposure] significantly decreased t- cholesterol, TGs, VLDL and LDL by 46.56%, 44.51%, 35.38%, and 50.98%, respectively, as compared to IR non-treated rats (*p* = 0.001).

**Table 1:** Effect of Ubq on serum t-cholesterol, TGs, VLDL, LDL and HDL in irradiated rats.

	t-cholesterol (mg/dl)	TGs (mg/dl)	VLDL (mg/dl)	LDL (mg/dl)	HDL (mg/dl)
Control	43.01 ± 2.79	199.3 ± 3.07	50.01 ± 4.91	85.44 ± 1.67	50.08 ± 2.89
IR	88.93 ± 4.60*	399.0 ± 3.62*	83.69 ± 5.33*	138.7 ± 4.63*	53.46 ± 3.92
IR + Ubq	47.52 ± 4.90^#^	221.4 ± 18.82^#^	54.08 ± 5.25^#^	67.98 ± 5.49*^#^	57.38 ± 1.27

Rats (n = 6) exposed to radiation (7 Gy) and received Ubq [10 mg/kg/day, p.o] 7 days pre- and 7 days post-radiation. Values are represented as mean ± S.E.M. Significant difference against control represented as (*) at p ≤ 0.05, significant difference against IR group represented as (#) at *p* ≤ 0.05, One-way ANOVA was carried using Graph pad prism software followed by Tukeyʾs Multiple Comparisons test.

### Assessment of serum 8-OHdG and MMP-9

**Figure 3:**
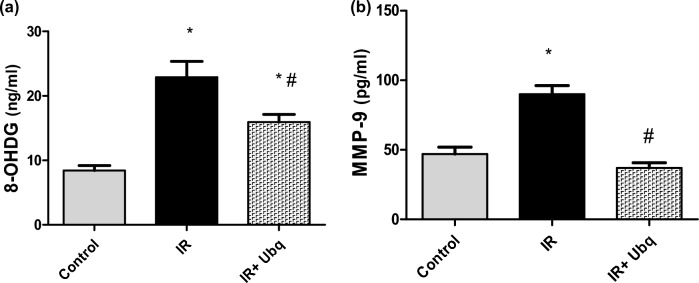
Levels of (**a**) 8-OHdG and (**b**) MMP-9 in serum of control, IR (7 Gy) and IR + Ubq (10 mg/kg/day) groups. Ubq was administered orally to rats (n = 6) for 7 days pre- and 7 days post-radiation. Data were expressed as mean ± S.E.M. Significant difference against control group represented as (*) at *p* ≤ 0.05, significant difference against IR group represented as (#) at *p* ≤ 0.05, One-way ANOVA was carried using Graph pad prism software followed by Tukeyʾs multiple comparisons test.

Exposure to γ-radiation led to marked increment in the serum level of 8-OHdG by 171.53%, as compared to control group (*p* = 0.001), reflecting considerable oxidative damage. Treatment with Ubq reduced the 8-OHdG level by 30.36%, as compared to IR group (*p* = 0.1) (Fig. 3a). Similarly, irradiation of rats induced a significant increase in serum MMP-9 by 91.40%, compared to normal value (*p* = 0.001), however, treatment with Ubq (10 mg/kg) significantly suppressed MMP-9 by 58.89 (*p* = 0.001), as shown in Fig. 3b.

### PCR quantification of ICAM-1 in aortic arch tissue

**Figure 4:**
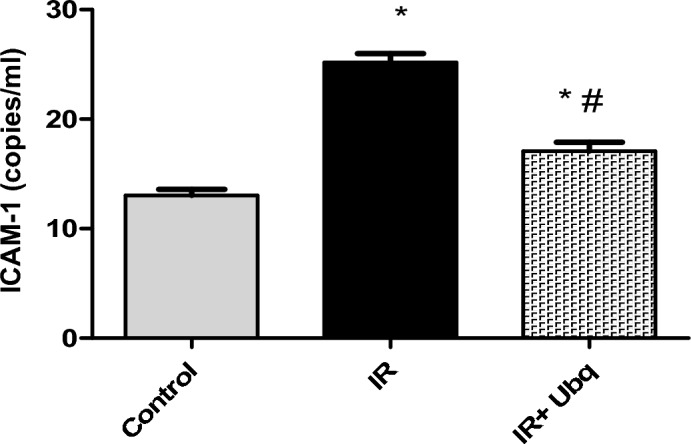
ICAM-1 expression (copies × 10^4^/ml) in aortic arch tissue of control, IR (7 Gy) and IR + Ubq (10 mg/kg/day) groups. Ubq was administered orally to rats (n = 6) for 7 days pre- and 7 days post-radiation. Data were expressed as mean ± S.E.M. Significant difference against control group represented as (*) at *p* ≤ 0.05, significant difference against IR group represented as (#) at *p* ≤ 0.05, One-way ANOVA was carried using Graph pad prism software followed by Tukeyʾs multiple comparisons test.

Acute exposure to γ-radiation (7 Gy) up-regulated the mRNA expression of ICAM-1 by 93.1%, as compared to control group (*p* = 0.001), which led to an established inflammatory reaction in aortic arch tissue. Treatment with Ubq for 14 days [7 days pre-irradiation and 7 days post irradiation] suppressed the mRNA expression of ICAM-1 by 32.22%, as compared to IR group(*p* = 0.001) (Fig. 4).

### PDGF and p38 MAPK determination in aortic arch tissue

**Figure 5:**
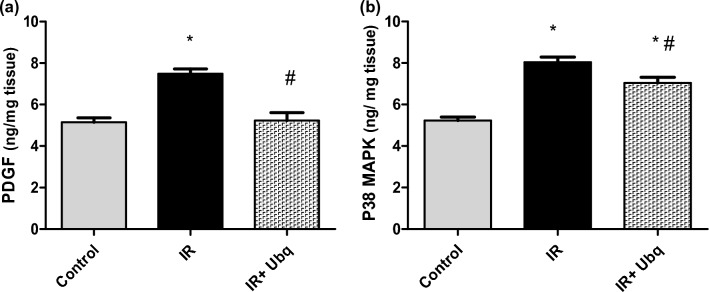
Levels of (**a**) PDGF (ng/mg tissue) and (**b**) p38 MAPK (ng/mg tissue) in aortic tissue of control, IR (7 Gy) and IR + Ubq (10 mg/kg/day) groups. Ubq was administered orally to rats (n = 6) for 7 days pre- and 7 days post-radiation. Data were expressed as mean ± S.E.M. Significant difference against control group represented as (*) at *p* ≤ 0.05, significant difference against IR group represented as (#) at *p* ≤ 0.05, One-way ANOVA was carried using Graph pad prism software followed by Tukeyʾs multiple comparisons test.

The aortic content of PDGF and p38 MAPK were significantly increased in irradiated rats by 45.59% (*p* = 0.001) and 53.72% (*p* = 0.001), respectively, as compared to control group (Fig. 5a,b). Treatment with Ubq pre- and post- radiation exposure significantly lowered the PDGF and p38 MAPK concentrations by 30.15% (*p* = 0.001) and 12.46% (*p* = 0.1), respectively, as compared to IR non-treated group (Fig. 5a,b).

## Discussion

Animal models using high-dose irradiation are in agreement with some of the human data from cancer therapy patients, highlighting the structural and functional alterations induced in the vasculature upon radiation exposure^3,4,23^. In the present study, exposure to radiation at a dose level of 7 Gy showed vascular endothelial damage in aortic arch and coronary artery tissues, as shown in the histopathological examination. These results agreed with a previous study by Mancuso, et al.^24^ showing that irradiation at a high dose level of 6 Gy was associated with atherosclerotic progression, which could be considered a chronic inflammatory state, starting with endothelial dysfunction that originates primarily from accumulation of ROS in arteries walls^25^. Treatment with Ubq, the reduced form of CoQ10, was capable of attenuating these morphological alterations and restoring endothelial function effectively. The first explanation comes to mind in this matter, is the manifested antioxidant potency of CoQ10^26^.

In consistence with the current histopathological results, the lipid profile data showed that exposure to 7 Gy gamma radiation increased the serum TC, TGs, LDL, and VLDL with no change in HDL. The alterations in these blood lipid fractions activate endothelial cells, which in turn express adhesion molecules and differentiate macrophages into foam cells, which is the first sign of endothelial dysfunction^27,28^. The elevation in serum lipid fractions might result from the ability of radiation to accelerate pathways of cholesterol formation like increasing its rate of biosynthesis in the liver and other tissues, as well as from the disturbance of LDL cholesterol receptors, leading to hypercholesterolemia, which has a particular effect on polyunsaturated fatty acids and increase lipid peroxidation^29^. Therefore, keeping the lipid profile under control might protect against vascular endothelial dysfunction. Administration of Ubq maintained the serum TC, TGs, LDL and VLDL nearly at normal levels which positively affect the endothelial function in the examined coronary and aortic wall. The present results agreed with Modi, et al.^30^ who showed that treatment with Coenzyme Q10 decreased serum cholesterol and serum triglyceride levels in diabetic rats.

Considering all of the aforementioned results, the current investigation was extended to verify the molecular route accounting for the Ubq’s protective impact against radiation-induced endothelial vascular harm.

ROS is the main key to realize the relationship between radiation and endothelial vascular injury; the presence of a large amount of water in myocardial tissue, decomposed during radiation exposure, is one of the main sources of ROS^31^. The imbalance between the oxidation and the antioxidant systems leads to ROS accumulation, cardiac injury and DNA damage. As a result of DNA being attacked by ROS, strand breaks and base modifications occur. 8-hydroxy-2-deoxy guanosine (8-OHdG), one of these base modification products, is used as an important indicator of oxidative DNA damage^32^. This might explain the dramatic increase in 8-OHdG level in the serum of irradiated rats, reflecting the DNA oxidative damage due to excessive production of ROS induced by gamma-radiation. In agreement with the current findings; El Adham et al.^33^ showed that level of 8-OHdG was increased in the serum of irradiated rats as a sign of damage provoked by exposure to gamma rays. Many studies suggested that high levels of 8-OHdG could be considered a serious risk factor of coronary and cardiovascular diseases^34–36^, an increase which resembles that observed in the current findings. Notably, the current study demonstrated that Ubq has a protective effect against DNA oxidative damage, as demonstrated by a reduction in the serum 8-OHdG level. Such effect was in line with Kaya et al.^37^, which revealed that low levels of endogenous CoQ10 are linked to DNA oxidative damage and have an adverse impact on coronary arteries.

Moreover, continuous exposure of LDL to substantial oxidative modifications induced by radiation may result in the formation of Ox-LDL, that appears to initiate complex inflammatory and immunologic mechanisms. This led to release of chemokines and foam cells formation that promote endothelial dysfunction^38^. The Ox-LDL was reported to promote more ROS production, macrophage proliferation, and subsequent platelet activations, while inhibiting the synthesis of endothelial nitric oxide synthase (eNOS)^28^. Additionally, it was found that lectin-type oxidized LDL receptor-1 (LOX-1), a scavenging receptor for Ox-LDL, was secreted in a dose dependent matter in response to radiation, this ligand-receptor binding is the starting step in endothelial cells dysfunction and apoptosis, as well as the formation of foam cells in vascular smooth cells^28,39^. Interestingly, CoQ10 is a well-known powerful lipophilic endogenous antioxidant that participates in the mitochondrial transport chain reaction, which can also suppress LOX-1 expression and inhibits more generation of ROS^40^, as well as, it prevents LDL oxidation and relieves endothelial dysfunction^20^.

Here attention needs to be paid to the fact that both ROS and Ox-LDL activate p38/MAPK signaling in endothelial cells^28^. Moreover, Xiao, et al.^9^ reported the relationship between MAPKs activation and exposure to radiation. p38 MAPK is one of mitogen activated serine/threonine kinase, and usually phosphorylated and activated by extracellular stressors like radiation, or intracellular one as in case of DNA damage, followed by activation of extracellular signal-regulated kinases (ERKs) and c-Jun N-terminal kinases (JNKs) pathways, which subsequently up-regulated pro-inflammatory mediators such as vascular adhesion molecules, E-selectin and NF-κB^41,42^. Furthermore, p38 MAPK has a principal role in expression of inflammatory mediators in macrophages which involved in cardiovascular, diabetic, nephropathy and arthritic complications^43^. The present results revealed that γ-radiation sharply increased the phosphorylation of p38 MAPK, which in turn, was inhibited by treatment with Ubq. These results agreed with those of Pérez-Sánchez, et al.^21^, where Ubq supplementation improved endothelial function and inhibited phosphorylation of thrombosis-related protein kinases as p38 MAPK in patients with antiphospholipid syndrome. Estimating the total p38 would validate the present findings; however, its omission was regarded as a research limitation.

In the current study, we further investigated the mechanisms underlying the inhibitory effect of Ubq on MAPK, in particular p38, induced by irradiation. One of the suggested pathways is interfering with PDGF signaling; the up-regulation of PDGF in response to irradiation was shown in the present findings and was in accordance with previous study of Bujak and Frangogiannis^44^. When PDGF is activated, it binds to its receptor, a receptor tyrosine kinase (RTK), which is phosphorylated to activate downstream signaling cascades, among which is MAPK that has been shown to regulate fibroblast growth^45,46^. Therefore, the activation of PDGF was one of the pathways that stimulate p38 MAPK signaling. Pretreatment with Ubq inhibited the expression of PDGF induced by irradiation, which in turn suppressed the p38 MAPK signaling.

Based on the consideration that cell migration is a process of periodical changes of cytoskeleton, p38 MAPK signaling is involved in cytoskeleton remodeling and thus involved in the regulation of cell migration^47^. Radiation-induced inflammation in the endothelium was associated with the expression of adhesion molecules in response to p38 MAPK signaling, which led to the formation of atherosclerotic plaques, thus forming an integrated and overlapping system for migration of leukocyte from circulation into the inflamed vascular wall^48^. That was verified in the current data as the activity of aortic ICAM-1 increased after exposure to 7 GY γ-radiation. Such effect was in agreement with study of Verma et al.^49^, showing that the level of ICAM-1, primarily expressed in the microvasculature increased after exposure to high doses of radiation. Therefore, the reduction of ICAM-1 caused by Ubq supplementation suggested that Ubq plays a crucial role in regulating endothelial dysfunction. Similarly, Mohseni et al.^50^ clearly demonstrated that CoQ10 supplementation can reduce the ICAM-1 and IL-6 serum concentration in patients with Myocardial infarction.

The proteolytic MMP-9 enzyme was also investigated in the current work, as it plays a critical role in the degradation of the extracellular matrix in response to atherosclerotic plaque formation^51^. Further, MMP-9 may also promote transmigration of monocytes across the endothelium and disrupt the basement membrane surrounding endothelial cells, which, may lead to destabilization of the plaque^52^. The expression of MMP-9 is up-regulated in response to radiation exposure^53,54^. These findings agreed with the current elevated MMP-9 activity noted after 7 days of irradiation. The foam cells as one of macrophages phenotype were considered a direct source of MMP-9^55^. Therefore, the activation of MMP-9 could be the reason of the edema in-between the elastic bands seen in the histological examination of irradiated rat arteries. On the other side, the current results showed that the induced MMP-9 activity in irradiated rats was reduced by Ubq supplementation. Similar results were obtained by Nattagh-Eshtivani et al.^56^ where CoQ10 supplementation was reported to significantly reduce MMP-9 levels in women with migraine.

**Figure 6:**
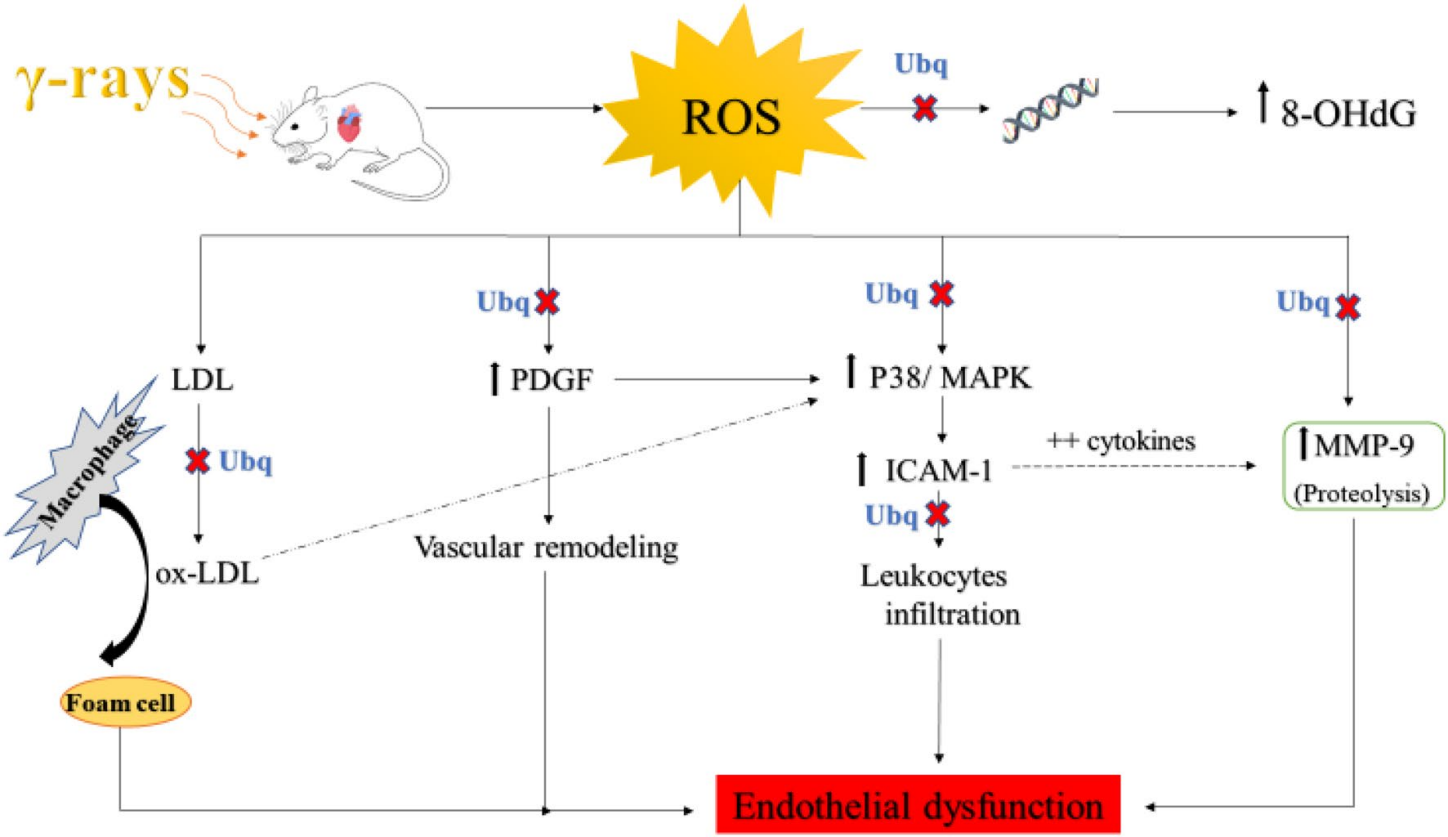
The suggested pathway for Ubq to hinder the detrimental effects of radiation exposure on the aortic arch and coronary artery. Rats were exposed to acute radiation dose level of 7 Gy. Ubq was administered orally to rats (n = 6) for 7 days pre- and 7 days post-radiation.

As a conclusion, Ubq significantly lessened the radiation-induced degrading effects on the coronary artery and aortic arch. This effect was verified by histopathological examination and biochemical investigations; Ubq restored the lipid profile, in particular LDL, and suppressed the oxidative DNA damage by combating ROS. Moreover, Ubq provided more endothelial protection by prompting the MMP-9 activity and interfering with the PDGF/p38 MAPK signaling, leading subsequently to reduction in ICAM-1 which regulate the leukocyte recruitment. The suggested pathway was clearly demonstrated in Fig. 6.

## Material and methods

### Animals

Male albino rats (200–230 g) brought from the animal house of National Center for Radiation Research and technology (NCRRT). Animals were accessed to standard pellet diet and water *ad* libitum.

### Drug

Ubiquinol: the reduced form of Ubiquinone (Co-Q 10) obtained from CanPrev^®^ [Premium Natural Health Products, Canada] in the form of 100 mg soft gel capsules. Each capsule was evacuated and dissolved in 1% tween 80 solution and administrated to rats at a dose of 10 mg/kg/day, p.o. according to Couto, et al.^57^.

### Irradiation of animals

Rats were exposed to whole body γ-radiation at an acute dose of 7 Gy^58^. Irradiation process was carried out at the Gamma Cell-40 biological irradiator (^137^Cs irradiator unit, Sheridan science and technology park, Mississauga, Ontario, Canada). The dose rate 0.33 Gy/min. at the time of experiment.

### Experimental design

#### A pilot study was carried out in two stages

The 1st stage was conducted using histopathological examination to select the optimum radiation interval that will induce vascular changes in the aortic arch and coronary artery. Rats were randomly allocated into 3 groups (n = 6); the first group (IR1) was exposed to a dose level of 7 Gy then euthanized after 7 days; the second group (IR2) was exposed to a dose level of 7 Gy then euthanized after 14 days and the third group was left non-irradiated (Normal). Based on the outcomes, the 7 days interval was selected.

In-order to select the most effective Ubq treatment plan, a second stage was conducted where another 18 rats were exposed to 7 Gy gamma irradiation and then allocated into three groups (n = 6). The 1st group received a vehicle of 1% tween 80 (IR), the 2nd group was orally treated with Ubq (10 mg/kg) daily for 7 days (Ubq post radiation), and the 3rd group received daily oral treatment of Ubq (10 mg/kg) for 7 days prior to irradiation and another 7 days post-irradiation (Ubq pre and post-radiation). According to the results, administration of Ubq pre- and post-radiation was selected.

Based on the pilot results, a set of 18 rats were allocated randomly into 3 groups (n = 6). The first group received vehicle of 1% tween 80 (Control). The second group was irradiated (7 Gy) and received vehicle of 1% tween 80 (IR). Rats in the 3rd group received Ubq (10 mg/kg/day, p.o.) for 7 days prior to irradiation and another 7 days post-irradiation (Ubq).

At the end of the experiment, all rats were anesthetized with urethane (1 g/kg, i.p), and then sacrificed by cervical dislocation. Blood samples were withdrawn from retro-orbital plexus, then centrifuged at 4°c for 15 min at 4000 rpm for obtaining serum samples, which stored at − 80 °C for assessment of total lipid profile (t-Cholesterol, TG, VLDL, LDL and HDL), matrix metalloproteinase (MMP-9) and 8-hydroxydeoxyguanosine (8-OHdG). The arch of aorta were rapidly excised, rinsed with ice-cold saline and stored in − 80 °C till being used for quantification of platelet derived growth factor (PDGF), p38 mitogen activated protein kinase (MAPK), as well as, gene expression of intercellular adhesion molecule (ICAM-1). For histopathological examination, aortic arch and heart samples were washed with saline and then embedded in 10% formalin until investigation.

### Histopathological evaluation and image acquisition

Coronary artery and aortic arch tissue specimens were fixed in 10% neutral buffered formalin. The fixed specimens were then trimmed, washed and dehydrated in ascending grades of alcohol, cleared in xylene, embedded in paraffin, sectioned at 4-6U thickness and stained by hematoxylin and eosin, according to Bancroft et al.^59^.

The sections were evaluated by scanning the entire tissue specimen under low-power magnification (×40) and then confirmed under higher power magnification (×200 and ×400). The severity of histopathological findings was scored as (0) absent, (1) minimal, (2) mild, (3) moderate and (4) marked change. All histopathological scoring and evaluation were carried out blindly. Images were obtained under a light microscope.

### Biochemical analysis

#### Serum lipid profile assessment [t-cholesterol, TG, VLDL, LDL and HDL]

Assessment of total cholesterol, LDL, HDL, TGs and VLDL levels in serum was carried by using specific colorimetric assay kits (Biodiagnostic Co., Egypt) and according to the manufacturer’s instructions. Values were expressed as mg/dl.

#### Serum 8-OHdG and MMP-9 assessment

ELISA kits were used for assessing of serum 8-OHdG (ng/ml) [8-OHdG ELISA kit, Cat. No. E-EL-0028, Elabscience^®^, USA] and serum MMP-9 (pg/ml) (MMP-9 ELISA kit, Cat. No. E-CL-R0449, Elabscience^®^, USA) according to the manufacturer’s instructions.

#### PCR absolute quantification of ICAM-1 in aortic arch tissue

Total RNA (2 μg) was extracted from aortic tissue using RNeasy Mini kit (Cat. No.: 61074, QIAGEN GmbH, Hilden, Germany), according to the manufacturer's instructions. Then, RNA quantification was done spectrophotometrically at 260 nm and 280 (Denovix DS-11 spectrophotometer). Isolated RNA was converted to cDNA (2 μl) by superscript first-strand synthesis kit (Thermo Fisher Scientific). Real-time PCR was conducted to assess the ICAM-1 expression using 2 μL RNA, Controls and references with a mixture containing 10 μL SYPR Green Master Mix and 0.63 μL Hot Start Taq RNA polymerase [Cat. No. 204074, SYBR Green/ ROX qPCR Master Mix Kit, Frmentas Life Science]. Based on the manufacturer’s guidelines, the following thermo-cycling conditions were applied in standard mode: the PCR mixture is incubated for 2 min at 50 °C then an initial denaturation is carried out at 95 °C for 10 min followed by 45 cycles of amplification: 95 °C for 10 s, 58 °C for 15 s and 72 °C for 20 s followed by a final step of 7 min at 72 °C. The real time PCR reaction was conducted in a Rotor-Gene Q5 plex real time Rotary analyzer (Corbett Life Science, USA). The nucleotide sequences of the primers are shown in Table 2. ABI prism R 7000 SDS software was used to analyze the data. Gene expression (i.e., number of copies) was calculated from the standard curve provided; serial tenfold dilutions of known quantities, ranging from 10 to 1 × 10^−4^ pg/µL corresponding to 4.20 × 10^22^–4.20 × 10^17^ Icam-1-copies/µL were made. The median of the RNA expression was calculated and used as a threshold to differentiate between the higher and lower expression within the factor groups. The mRNA expression was normalized to the expression of the house keeping gene glyceraldehyde 3-phosphate dehydrogenase (GAPDH)^60,61^.

**Table 2:** Primer sequences.

ICAM-1_Forward_	5-AAACGGGAGATGAATGGTACCTAC-3
ICAM-1_Reverse_	5-TGCACGTCCCTGGTGATACTC-3
GADPH_forward_	5′-CCTACCCCCAATGTATCCGTTGTG-3′
GADPH_Reverse_	5′-GGAGGAATGGGAGTTGCTGTTGAA-3′

#### PDGF and p38 MAPK determination in aortic arch tissue

ELISA kits were used for assessing the aortic tissue concentrations of PDGF [PDGF ELISA kit, Cat. No. ER1352, Fine Test^®^, China] and p38 MAPK [MAPK (p38 Mitogen Activated Protein Kinase) ELISA kit, Cat. No. ER1191, Fine Test^®^, China] according to the manufacturer’s instructions. Values were expressed as ng/mg.

### Statistical analysis

Data were expressed as mean ± standard error mean (SE). Statistical analysis was carried out using one-way analysis of variance (ANOVA) test followed by Tukey- Kramer multiple comparison’s post-test. GraphPad Prism^®^ software package, version 6 (GraphPad Software Inc., USA) was used to carry out all statistical tests.

### Ethics approval

All animal experiments complied with the Animal Research Reporting of In-Vivo Experiments (ARRIVE) guidelines and were carried out in accordance the National Research Council's Guide for the Care and Use of Laboratory Animals (NIH publications No. 8023, revised 1978). The in-vivo study and all the methods were performed according to the guidelines set by the Ethics Committee at the NCRRT (permit number: 20 A/22).

## Data Availability

The data that support the findings of this study are available from the corresponding author upon request.
